# SSVEP Response Is Related to Functional Brain Network Topology Entrained by the Flickering Stimulus

**DOI:** 10.1371/journal.pone.0072654

**Published:** 2013-09-09

**Authors:** Yangsong Zhang, Peng Xu, Yingling Huang, Kaiwen Cheng, Dezhong Yao

**Affiliations:** Key Laboratory for NeuroInformation of Ministry of Education, School of Life Science and Technology, University of Electronic Science and Technology of China, Chengdu, China; National Research & Technology Council, Argentina

## Abstract

Previous studies have shown that the brain network topology correlates with the cognitive function. However, few studies have investigated the relationship between functional brain networks that process sensory inputs and outputs. In this study, we focus on steady-state paradigms using a periodic visual stimulus, which are increasingly being used in both brain-computer interface (BCI) and cognitive neuroscience researches. Using the graph theoretical analysis, we investigated the relationship between the topology of functional networks entrained by periodic stimuli and steady state visually evoked potentials (SSVEP) using two frequencies and eleven subjects. First, the entire functional network (Network 0) of each frequency for each subject was constructed according to the coherence between electrode pairs. Next, Network 0 was divided into three sub-networks, in which the connection strengths were either significantly (positively for Network 1, negatively for Network 3) or non-significantly (Network 2) correlated with the SSVEP responses. Our results revealed that the SSVEP responses were positively correlated to the mean functional connectivity, clustering coefficient, and global and local efficiencies, while these responses were negatively correlated with the characteristic path length of Networks 0, 1 and 2. Furthermore, the strengths of these connections that significantly correlated with the SSVEP (both positively and negatively) were mainly found to be long-range connections between the parietal-occipital and frontal regions. These results indicate that larger SSVEP responses correspond with better functional network topology structures. This study may provide new insights for understanding brain mechanisms when using SSVEPs as frequency tags.

## Introduction

The human brain is a large-scale complex network composed of widely distributed, functionally specialized, and interconnected brain regions. In recent years, graph theories have been introduced to study the functional connectivity of the brain, which allows us to elucidate the relationship between the topological structure of brain networks and processes occuring in those networks [1]. When applied to brain research, these approaches offer a network perspective that help us better understand the higher brain functioning [2], [3], [4], [5]. Previous studies have suggested that the brain network topology correlates with its cognitive function. A resting-state fMRI study indicated that a strong positive association exists between the intellectual performance and the global efficiency of brain networks [4]. Furthermore, a resting-state EEG study showed a correlation between a shorter reaction time and a shorter characteristic path length within gamma band network [6]. Functional connectivity analysis is assumed to provide new avenues to measure and assess the functional interactions between brain regions. Few studies have investigated the relationship between sensory functional brain networks and their outputs. Sensory functional brain networks can be investigated using steady-state paradigms with a periodic stimulus. These studies provide us with new clues regarding the relationship between steady state visual evoked potentials (SSVEP) and the corresponding network topology.

SSVEP is a periodic response to a regularly repetitive visual stimulus modulated at a fixed frequency larger than 4 Hz [7]. It is actually a near-sinusoidal waveform that contains the same fundamental frequency of the visual stimulus and its harmonics. SSVEP responses have a stable spectrum and a high signal-to-noise ratio, and they can be evoked by a visual stimulus with a flickering frequency ranging between 4 and 75 Hz [8]. As a frequency-tagging method, SSVEP is a very useful tool for the study of neural processes underlying brain rhythmic activities, and it has been successfully used in a wide range of applications in cognitive neuroscience and clinical researches [9]. Moreover, SSVEP is also used in the brain computer interface (BCI) to achieve a high information transfer rate [10], [11], [12], [13], [14], [15], [16], [17], [18]. A universal phenomenon especially observed in implementing a BCI is that different subjects obtain different performances using the same SSVEP-BCI systems [10], [11], [12], [19]. This phenomenon may indicative of variability between interindividual in SSVEP responses under the same periodic stimuli.

Evidences suggest that the SSVEP response involves multiple sources distributed cortical regions, including the parietal, temporal, frontal, and prefrontal cortices [9], [20]. Additionally, functional networks including these regions are sensitive to the physical properties of the stimulus, especially to the driving frequencies [20]. The amplitude, phase and spatial distribution of SSVEP responses change with the flickering frequencies. As a whole, this indicates that different frequencies can target functionally distinct brain networks with different preferred or resonant frequencies [7], [20]. However, very few studies have examined the functional network organizations underlying SSVEP. Using correlation analysis of fMRI data, Srinivasan et al. showed that occipital voxels were either positively or negatively correlated to frontal voxels forming functionally distinct large-scale functional networks [20]. In an EEG study, Yan et al. showed that the parietal cortical regions were a critical node for the transmission of SSVEP information [21]. To the best of our knowledge, the relationship between the network topological properties and SSVEP responses have not yet been examined.

Our motivation for the present study is to probe the association between SSVEP and the overall topology of the network entrained by the flickering stimulus using the graph theoretical analysis. Two frequencies flickering (see Materials and Methods) were used for the visual stimulus. During our experiment, the subjects were not required to conduct mental tasks.

## Materials and Methods

### Ethics Statement

This study was approved by the Institution Research Ethics Board at the University of Electronic Science & Technology of China. All participants were asked to read and sign an informed consent form before participating in the study. All the participants received monetary compensation for their time and effort following completion of the experiment.

### Participants

Eleven healthy right-handed adult volunteers (males; 24–27 years; mean 25 years) participated in these experiments. All of the subjects had normal or corrected-to-normal vision. These subjects did not have any history of epileptic seizure or mental disease.

### Flickering Stimuli

We used two stimulus cycles of 80 ms and 60 ms to generate the stimulus with two light-emitting diodes (LED). Therefore, two frequencies, 12.5 Hz and 16.6 Hz, were used in this experiment. Each LED was fixed at the end of a white pipe with a diameter of about 3 cm and a length of about 60 cm. The subjects gazed at the stimulus at the other end of the pipe. The background luminance was 0.25 cd/m^2^, the maximum luminance of the LED was 2.5 cd/m^2^, and the modulation depth was 82%. All the driving pulses used here had a 50% on/off duty cycle. These settings have been used in previous experiments [22], [23]. During the experiment, each subject completed two runs corresponding to each of the two frequencies. Each run had a duration of 2 mins, during which EEG data were recorded. In each run, the frequency was randomly assigend and presented to the subjects. Between runs, the subjects were allowed to rest for 2–5 minutes. In the experiment, the subjects were required to avoid blinking and large movements. The total duration of the experiment was about 40 minutes.

### EEG Acquisition

The experiment was conducted in a shielded room. The EEG data were recorded with a 129-channel EGI200 recording system (Electrical Geodesics Incorporated, USA). The 129 channels (128 measuring electrodes and 1 reference electrode Cz) included the international 10–20 standard recording electrodes. Electrode impedances were maintained below 10 kΩ during the experiment. EEG signals were digitized at 250 Hz, filtered online using a 0.3–70 Hz bandpass filter, and then stored on a disk for off-line analysis.

Electrodes with a preponderance of noise resulting from insufficient contact with the scalp, were excluded for analysis. A total of twenty-nine electrodes, primarily on the outer ring of the electrode array, were eliminated from the study due to excessive artifacts, leaving 100 electrodes [24], [25]. Additionally, data from other electrodes for which the original EEG was believed to be invalid (e.g., amplitude in excess of 100 µV), were replaced by the mean value of the three nearest recording sites [23]. For each subject, a total of 9–12 6 s –long segments of data free of artifacts (e.g., eye blinks, eye movements and muscle activities) were selected for each frequency. Following preprocessing, all data were re-referenced to zero reference by the reference electrode standardization technique (Free software: REST: doi:10.1016/j.clinph.2010.03.056, or www.neuro.uestc.edu.cn/rest/index.asp) [24], [26].

### Brain Network Construction

As closely spaced electrode pairs are subject to significant volume conduction effects [25], [27], 18 standard electrodes positions Fp1, Fp2, F7, F3, Fz, F4, F8, T3, C3, C4, T4, T5, P3, Pz, P4, T6, O1, O2 were chosen as the nodes for network construction. These electrodes covered all the regions that were assumed to be the possible sources of SSVEP [9], [20].

SSVEP can be measured in a narrow (usually<0.1 Hz) frequency band centered on the stimulus frequency; therefore, the coherence is adopted to measure the functional connectivity between each pair of electrodes. It is known that coherence is the most commonly used measure in the analysis of co-operative, synchrony-defined cortical neuronal assemblies [24], [28]. Coherence represents the linear relationship at a specific frequency between two signals *x*(*t*) and *y*(*t*), which is expressed as.
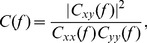
(1)where *C_xy_*(*f* ) is the cross-spectrum between *x*(*t*) and *y*(*t*), and *C_xx_*(*f* ), *C_yy_*(*f* ) are the auto spectra.

For each subject, we used a 2.4 s window length and a 1.2 s overlap to extract data epochs for all the data segments at each frequency. These data lengths were chosen because they included the integer number of cycles at all the frequencies. A narrow band spectrum with one bin centered on the stimulus frequencies was obtained for each segment. We then calculated the coherence matrix in each epoch and obtained a total of 36–48 coherence matrixes (of size 18 by 18) for each frequency. All the matrices of each frequency were averaged and used as the original whole network of each frequency.

The coherence was always nonzero, which might result in some spurious edges in the networks. Therefore, we tried to reduce these spurious edges using the sparsity value. Sparsity is the ratio of the total number of edges in a network divided by the maximum possible number of edges. A sparsity value can be obtained based on the criteria that all brain networks are fully connected while minimizing the number of false-positive paths [5], [29]. With this method, the resulting networks have the same numbers of nodes and edges [5], [29], and only retain edges with large connection strengths. In this study, the sparsity value was generated based on all original entire networks of two frequencies across 11 subjects. Because the sparsity value ranged from 0 to 1, we scaled the threshold value from 0 to 1 with a step of 0.01 to find the sparsity value. Under each threshold, after calculating the number of edges remaining in each network, we selected only the edges having the top connection strengths and deleted the rest edges. If a threshold reached the first value under which one of the original entire networks had a sole node, the sparsity value was the preceding threshold before that value.

### Brain Network Classification

After reducing a number of spurious connections based on the sparsity value, we denoted the new whole functional network as Network 0. To identify the connections (links) that were significantly correlated with the SSVEPs of each frequency, the correlation coefficient was calculated between the connection strengths of each possible nodal pair in Network 0 and the SSVEP responses across subjects. For example, we obtained 11 values of connection strengths between O1 and O2, and another 11 values of SSVEP responses under 12.5 Hz. Then, we calculated the correlation coefficient between these two groups of measurements to determine whether the connections between O1 and O2 were significantly correlated with the SSVEP responses under 12.5 Hz. With this approach, we found that the connections were significantly correlated with SSVEP responses of each frequency. We then divided each Network 0 into three sub-networks: Networks 1, 2 and 3. Network 1 was composed of connections in which the connection strengths were significantly positively correlated with SNRs (*p*<0.05, uncorrected). In Network 3, the connection strengths were significantly negatively correlated with SNRs. Finally, Network 2 was composed of the remaining connections for which the strengths were not significantly correlated with SSVEP responses. As the connections in Network 3 were very sparse, we did not calculate its network properties.

### Graph Theoretical Analysis

In this study, we focused on the weighted network analysis. In weighted networks, the weights indicate the connection strengthand reflect a difference in the capacity and intensity of the connections between nodes; thus, they may be a more valid approach for brain network modeling. Furthermore, using weighted networks is useful for reducing the influence of weak and potentially non-significant connections [5], [30].

In a weighted network (*N*-by-*N*), the clustering coefficient of a node *i* represents the likelihood that the direct neighbors of the node are also connected with each other [31], and it is calculated as follows:
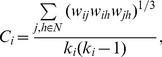
(2)where *w_ij_* is the weight between nodes *i* and *j* in the network, and *k_i_* is the degree of node *i*. In this work, *w_ij_* is the coherence between two electrodes obtained using formula (1).

The network clustering coefficient is the average of the clustering coefficients of all nodes:
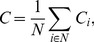
(3)it is a measure of the extent of the local density or cliquishness of the network [32].

The length of each edge is defined as the inverse of the edge weight, 1/*w_ij_*. The shortest path length between two nodes, *L_ij_*, is defined as the length of the path with the shortest length between the two nodes *i* and *j*. The characteristic path length of a network is measured by the harmonic mean length between pairs [33], to handle the possible disconnected edges and is calculated as follows:
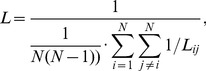
(4)this property quantifies the level of global communication efficiency of a network.

The global efficiency is defined by the inverse of the harmonic mean of the shortest path length between each pair of nodes [34], and it is computed as.
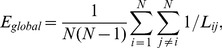
(5)it is a measure of the global efficiency of parallel information transfer in the network.

We can also compute the local efficiency of node *i* using the following formula:
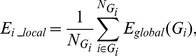
(6)where 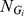 is the number of nodes in *G_i_* and *G_i_* denotes the subgraph composed of the set of nodes that are the direct neighbors of node *i* [34]. The mean local efficiency of graph *G* is the average of the local efficiencies of all nodes in graph *G*,



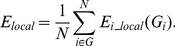
(7)It can be understood as a measure of the fault tolerance of the networks [35].

Graph theoretical analyses were implemented using the Brain Connectivity Toolbox [30].

### Relationship between Topological Properties and SSVEP

To reduce the influence of non-specific background, we expressed the SSVEP responses as signal-to-noise ratio (SNR) based on the Fourier transformation [36]. The SNR was defined as the ratio of the power of the stimulus frequency divided by the mean power value of the 1 Hz band that was centered on the stimulus frequency but excluded the stimulus frequency itself. The SNRs of the 18 electrodes of each frequency were averaged across the epochs for each subject. To avoid arbitrary selection of electrodes, we used the mean SNR of the 18 electrodes as a measure of the SSVEP responses of each frequency for each subject. We then correlated the network topological properties with the SNRs. The Pearson’s correlation analysis was used in this study.

## Results

Based on the original entire networks of the two frequencies from all the subjects, the sparsity value was calculated as 0.67. Before computing the topological properties, we first used this value to reduce the spurious edges of all the original whole networks.

### SSVEP Responses of Different Subjects Under the Two Frequency Stimuli

**Figure 1:**
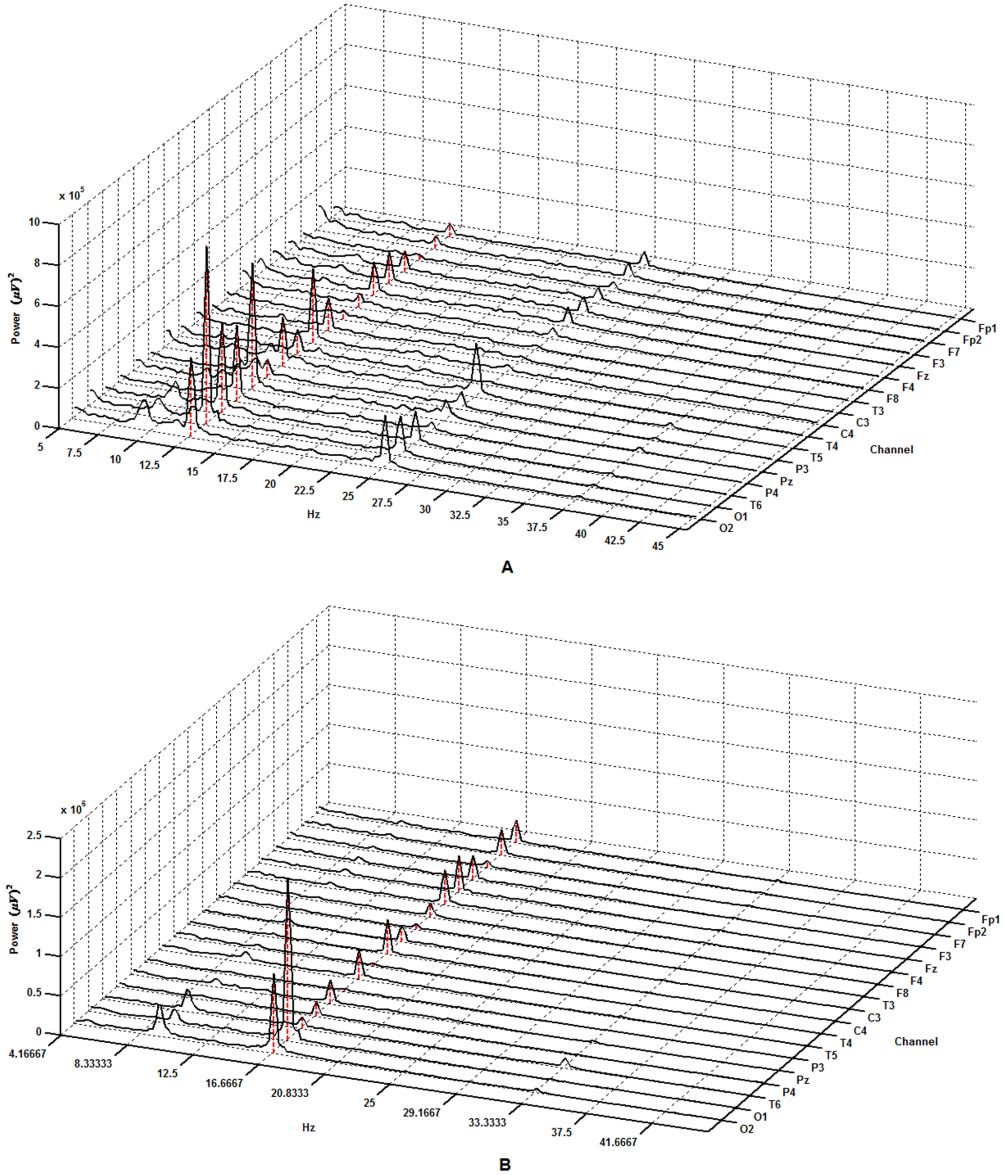
Power spectrum of eighteen electrodes averaged across the epoch from a subject. (A) 12.5 Hz, (B) 16.6 Hz. The red vertical lines indicate the components of the stimulus frequency.

**Figure 2:**
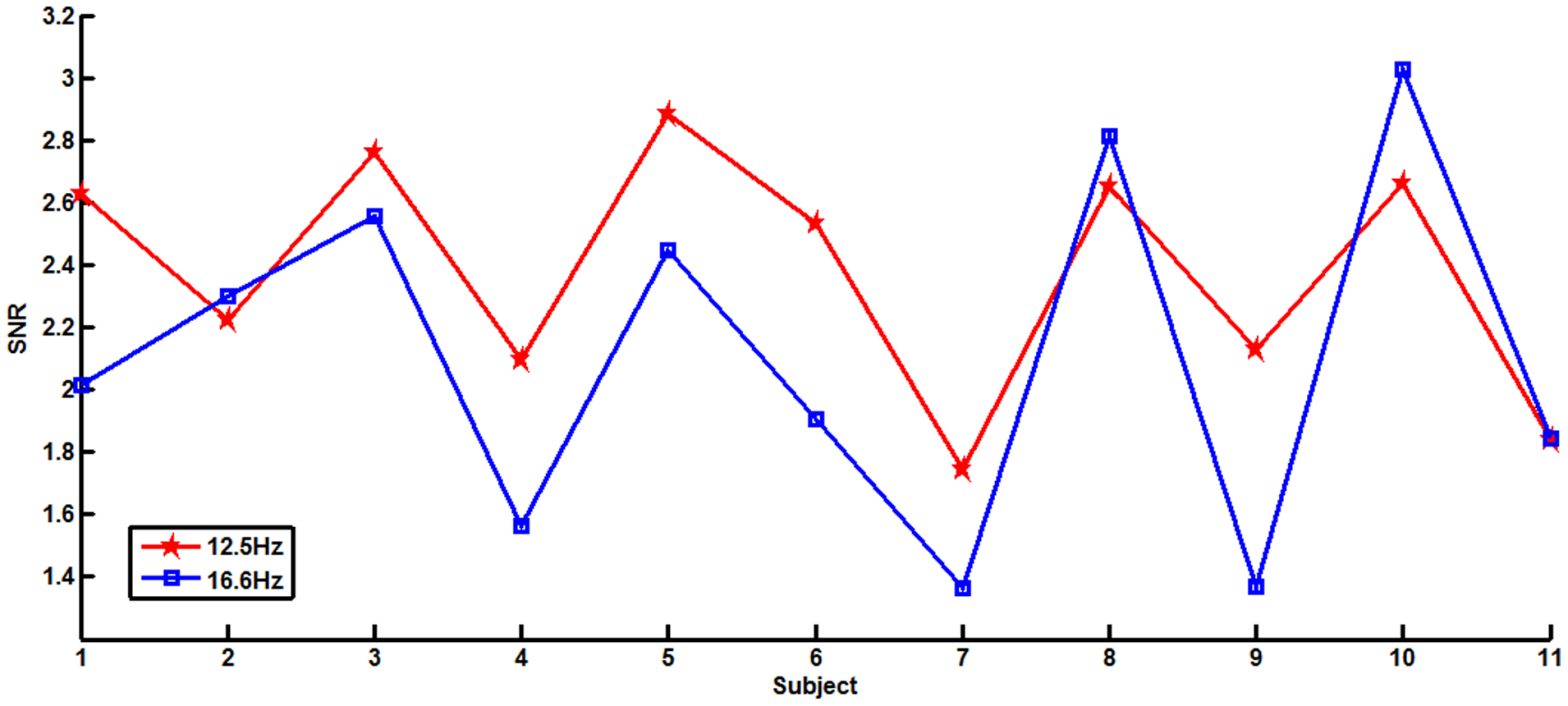
The SNRs of the two frequencies of different subjects.

To ensure that the experiment resulted in expected SSVEP responses, we calculated the power spectrum of each frequency for each subject. Two examples of power spectrum of both frequencies from a subject are shown in Figure 1. For this subject, the fundamental frequency components on eighteen channels were apparent under both stimulus frequencies. In addition, the second harmonic components were also apparent for most of channels under 12.5 Hz (Fig. 1(A)), while fewer harmonic components were found under 16.6 Hz (Fig. 1(B)). The patterns for the power spectrum varied between subjects, but all subjects displayed the expected evoked components for the two frequencies. At the same time, the mean SNRs averaged across the epochs and channels of all subjects, under both stimulus frequencies, are shown in Fig. 2. In this figure, different subjects showed variation in SNRs under the same frequency. These phenomena were present at both frequencies.

### Network Partition based on the Relationship between SNRs and Connection Strengths across Subjects

**Figure 3:**
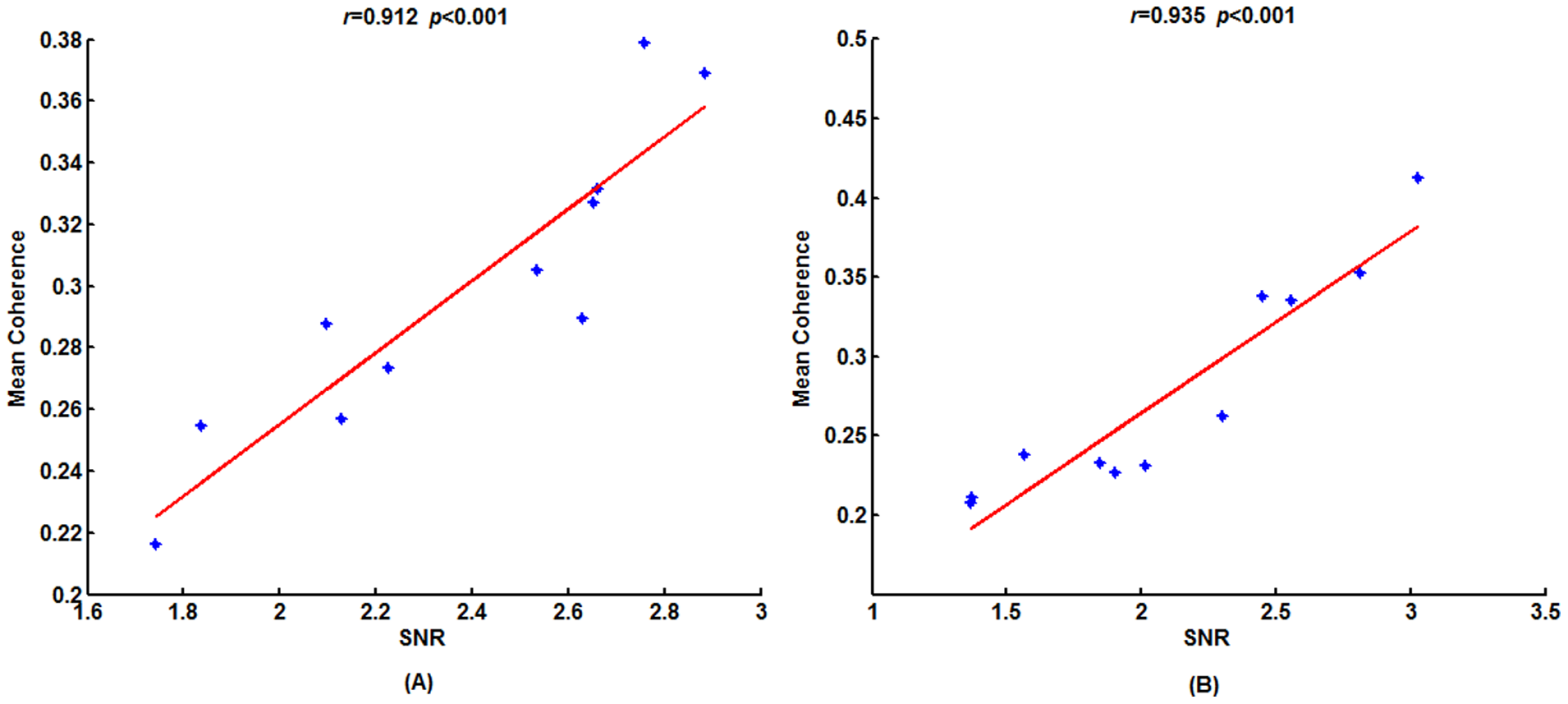
The correlation between the mean functional connectivity of Network 0 and SNRs across subjects. (A) 12.5 Hz, (B) 16.6 Hz. The red lines indicate the fitted linear trend. The *r* denotes correlation coefficients, *p* denotes significant level of correlation coefficients.

**Figure 4:**
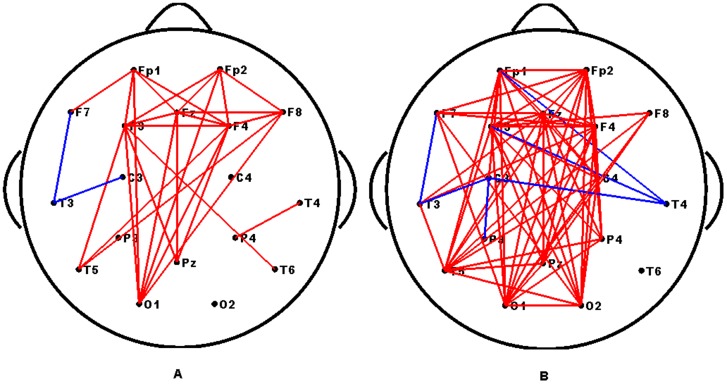
Spatial topology of links that showed significant correlation with SNRs of two frequencies. (A) 12.5 Hz, (B) 16.6 Hz. The red and blue lines indicate the links significantly positively or negatively correlated with the SNRs, respectively.

**Figure 5:**
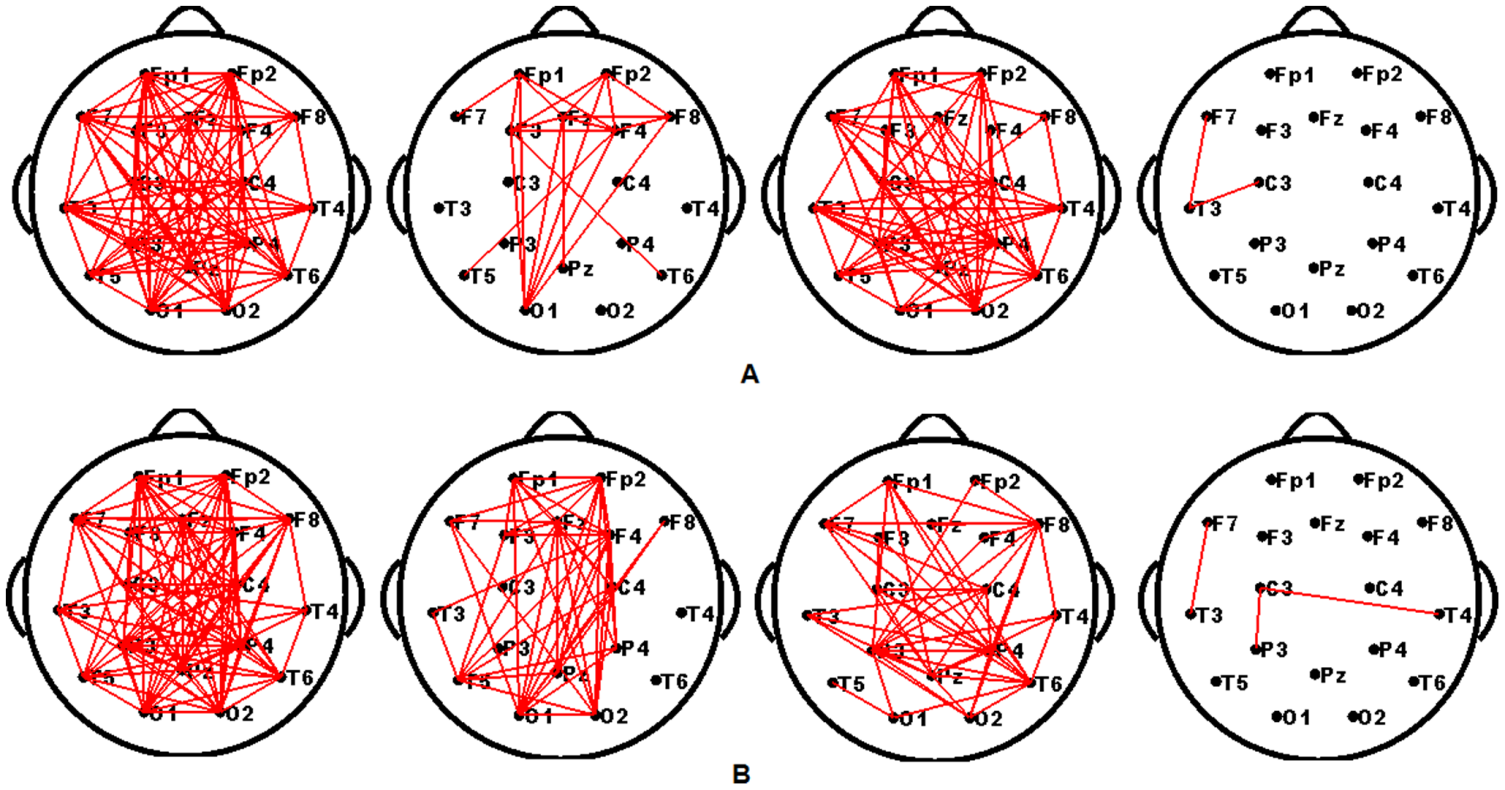
Four sub-networks’ topographies of one subject. For each row, from left to right, the four topology networks are Network 0, 1, 2 and 3, respectively. (A) 12.5 Hz, (B) 16.6 Hz.

As shown in Fig. 3, the mean functional connectivity of Network 0 is significantly correlated with the SNRs across subjects. This relationship may imply that both the SNRs and connection strengths are entrained by the stimulus, which results in a high correlation. To verify whether all connections were correlated with the SNRs, the correlation coefficient was calculated between the connection strengths of each edge present in Network 0 and the SNRs across subjects under each stimulus frequency. The computing procedure can be found in Section **“Brain network classification”.** Interestingly, we found that just a subset of the connections in Network 0 showed a positive correlation with the SNRs, and while an additional subset did not correlate with the SNRs. Additionally, there were also some sparse connections that correlated negatively with the SNRs. The connections that correlated significantly (*p*<0.05, uncorrected) with the SNRs are showed in Fig. 4. This result indicates that the significant links were mostly long-range connections between the occipital and frontal regions. Furthermore, according to the rules detailed in section **“Brain network classification”**, each Network 0 was divided into three sub-networks, i.e., Networks 1, 2 and 3. A representative example of these divisions were in Fig. 5.

Similar to Fig. 3 of Network 0, the mean functional connectivity at each frequency was also calculated for Networks 1 and 2 of each subject (Table 1). The mean functional connectivities of all three networks were found to be significantly correlated with SNRs. Taken as a whole, we may infer that the main contribution for SSVEP generation may not be simply attributed to connections entrained by stimulus; in other words, Network 1 is not the only contributor to the generation of SSVEP.

**Table 1:** The relationship between the mean functional connectivities of Networks 1 and 2 and the SNRs across subjects.

Network	Frequency	*r*	*p*
Network 1	12.5 Hz	0.892	***
	16.6 Hz	0.927	***
Network 2	12.5 Hz	0.875	***
	16.6 Hz	0.832	***

*r* denotes correlation coefficients, *p* denotes significant level of correlation coefficients.

*** *p*<0.001,

** *p*<0.01.

### Relationship between the SNRs and Topological Properties across Subjects

**Figure 6:**
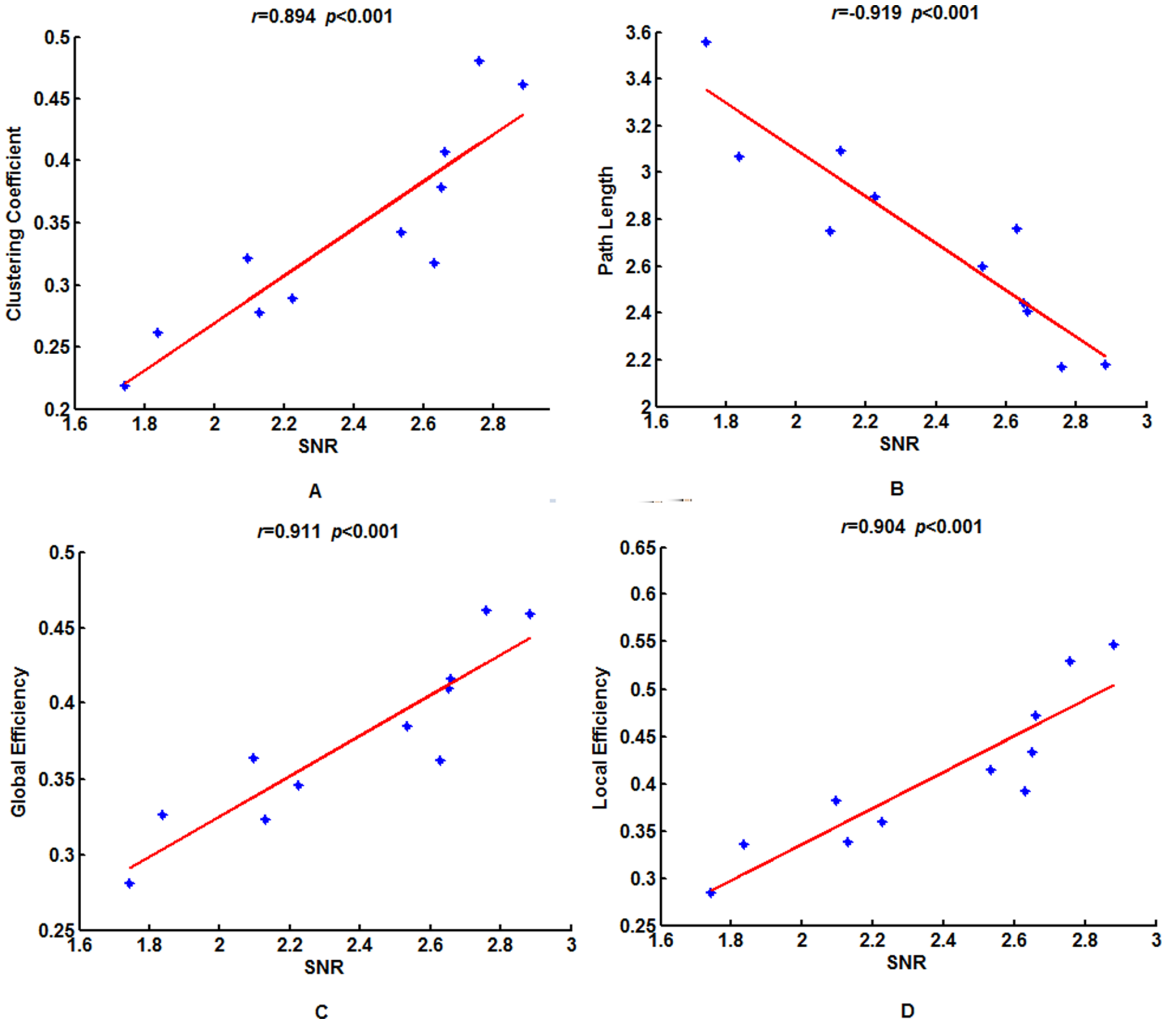
The correlations between the SNRs across subjects and the four network properties of Network 0 of 12.5 Clustering coefficient (A), global efficiency (C) and local efficiency (D) were significantly positively correlated with SNR, but the characteristic path length (B) was significantly negatively correlated with SNR. The red lines indicated the fitted linear trend. *r* denotes correlation coefficient, *p* denotes significant level of the correlation coefficient.

First, the four properties of Network 0 were calculated for each subject. At each frequency, we found strong associations between the SNRs and the topological properties (Fig. 6 and Table 2). The SNRs were significantly positively correlated with the clustering coefficient, global efficiency and local efficiency, and they were significantly negatively correlated with characteristic path length. Similarly, we calculated the four properties of Networks 1 and 2, and we found that the same relationships were present between the properties of these two sub-networks and SNRs (Tables 3 and 4). These results suggest that SNRs are likely to be related to the topology of individual functional brain networks with the same resonance frequency as the input stimulus frequency. Larger SSVEP SNRs are likely to correlate with more efficient functional brain networks. According to these results, it seems that the non-significant connections also contribute greatly to SSVEP generation, and as do their network topological properties.

**Table 2:** The relationship between network topological properties of Networks 0 and the SNRs of two flickering frequencies.

	Clustering Coefficient		Path Length		Global Efficiency		Local Efficiency	
Frequencies	*r*	*p*	*r*	*p*	*r*	*p*	*r*	*p*
12.5 Hz	0.894	***	−0.919	***	0.911	***	0.904	***
16.6 Hz	0.944	***	−0.9507	***	0.938	***	0.940	***

*r* denotes correlation coefficients, *p* denotes significant level of correlation coefficients.

*** *p*<0.001.

**Table 3:** The relationship between topological properties of Networks 1 and the SNRs of two flickering frequencies.

	Clustering Coefficient		Path Length		Global Efficiency		Local Efficiency	
Frequencies	*r*	*p*	*r*	*p*	*r*	*p*	*r*	*p*
12.5 Hz	0.842	***	−0.903	***	0.903	***	0.738	**
16.6 Hz	0.949	***	−0.947	***	0.924	***	0.960	***

*r* denotes correlation coefficients, *p* denotes significant level of correlation coefficients.

*** *p*<0.001,

** *p*<0.01.

**Table 4:** The relationship between topological properties of Networks 2 and the SNRs of two flickering frequencies.

	Clustering Coefficient		Path Length		Global Efficiency		Local Efficiency	
Frequencies	*r*	*p*	*r*	*p*	*r*	*p*	*r*	*p*
12.5 Hz	0.834	***	−0.898	***	0.890	***	0.871	***
16.6 Hz	0.801	**	−0.919	***	0.923	***	0.742	**

*r* denotes correlation coefficients, *p* denotes significant level of correlation coefficients.

*** *p*<0.001,

** *p*<0.01.

## Discussion

Graph theories have recently been widely employed to discover the underlying neural mechanisms of some neurological or cognitive phenomena [1], [4], [5], [29], [37].These findings may have important implications and applications, such as serving as the potential biomarkers for the observation of the diseases and cognitive function [5]. In this study, we attempted to apply graph theories analysis to explore functional brain networks of SSVEP and to identify the observed differences between subjects. To the best of our knowledge, this is the first study to investigate the relationship between SSVEP and brain networks that are entrained by a flickering stimulus.

### Relationship between SSVEP Responses and Functional Brain Networks

Studies have reported the resonant nature of SSVEP [38], [39], [40]. Neural circuits within the brain work at specific preferred or resonant frequencies, and the resonance properties of the EEG oscillators can be enhanced by the flickering stimulus [39]. These resonance phenomena may arise from either feedback connections within local neural circuits or the facilitation that arises through simultaneous stimulation of aggregates of similar types of neural circuits across the cortex [40]. Therefore, the flickering stimulus drives the brain neural circuits (oscillators) to shape the SSVEP brain networks. In our study, we found that the mean functional connectivity and topological properties of the networks (Networks 0) were correlated with the SNRs. We also obtained the same results with the sub-networks (Network 1) in which connections had a significantly positive correlation with the SNRs. Interestingly, in the sub-network (Network 2) in which connections were not correlated with SNRs, the overall mean functional connectivity and properties of the network were correlated with SNRs. Thus, the network that supports the SSVEP responses are complex and includes both connections that are directly correlated and uncorrelated with the SSVEP generation.

In addition, SSVEP may be quite possibly related to network efficiency. Within Networks 0, 1 and 2, we found the same relationships between the four topological properties and the SNRs. A shorter characteristic path length and a higher global efficiency of the network indicate more efficient parallel information transfer and integration in the brain, A larger clustering coefficient and local efficiency indicate larger local information processing. This suggests that larger SSVEP responses correspond to more efficient network organizations. The different topological brain networks sharing the same preferred frequency as the stimulus may act to modulate the same inputs to shape different response outputs. Our findings suggest that the efficiency of brain functional organization may be an important basis for SSVEP generation. These results may also be related to the observed phenomena that some subjects, using the same SSVEP-BCI system, do not perform as well as others. In the future, we need to further confirm these results using simultaneous EEG-fMRI data. These results may provide possible evidence for a significant role for complex brain network topological parameters have important value in understanding the neural mechanism of SSVEP.

In fact, some previous studies have shown similar associations between the brain network topology and some measurements of brain function. Li and his colleagues showed that individual differences in intelligence are associated with brain structural organization and that higher scores on intelligence tests were related to greater global efficiency of the brain anatomical network [37]. Another study also showed that a strong positive correlation exists between the global efficiency of resting-state functional brain networks and intelligence [4]. Zhou et al. showed that a shorter reaction time was correlated with a shorter characteristic path length in gamma band network using resting-sate EEG [6]. Douw et al. showed increased local connectivity in delta, theta and gamma bands was correlated with better cognition in a study using resting-state MEG [41]. The present study may add to the findings of these tpyes of studies.

### The Networks Comparisons between Two Groups of Subjects with High and Low SNR

To further explore the differences across subjects, we conducted a comparison study. We chose subjects with the four highest SNR (Group 1) and the four lowest SNR subjects (Group 2) to compare the nodal connectivity (*w_ij_*) differences. All the comparisons were performed using permutation tests [5], [42]. Accordingly, we calculated the actual differences of nodal connectivity (*w_ij_*) of Networks 0 of these groups. Next, each subject was randomly reassigned to one of the two groups of the same size as the original groups, and the connectivity differences were recalculated. This procedure was repeated 5000 times to sample the permutation distribution of connectivity differences under the null hypothesis that observed differences were not determined by the true group membership. The one-tailed *p* value was then computed as the proportion of differences in the permutation distribution that was greater (or smaller than) the observed between-group differences.

**Figure 7:**
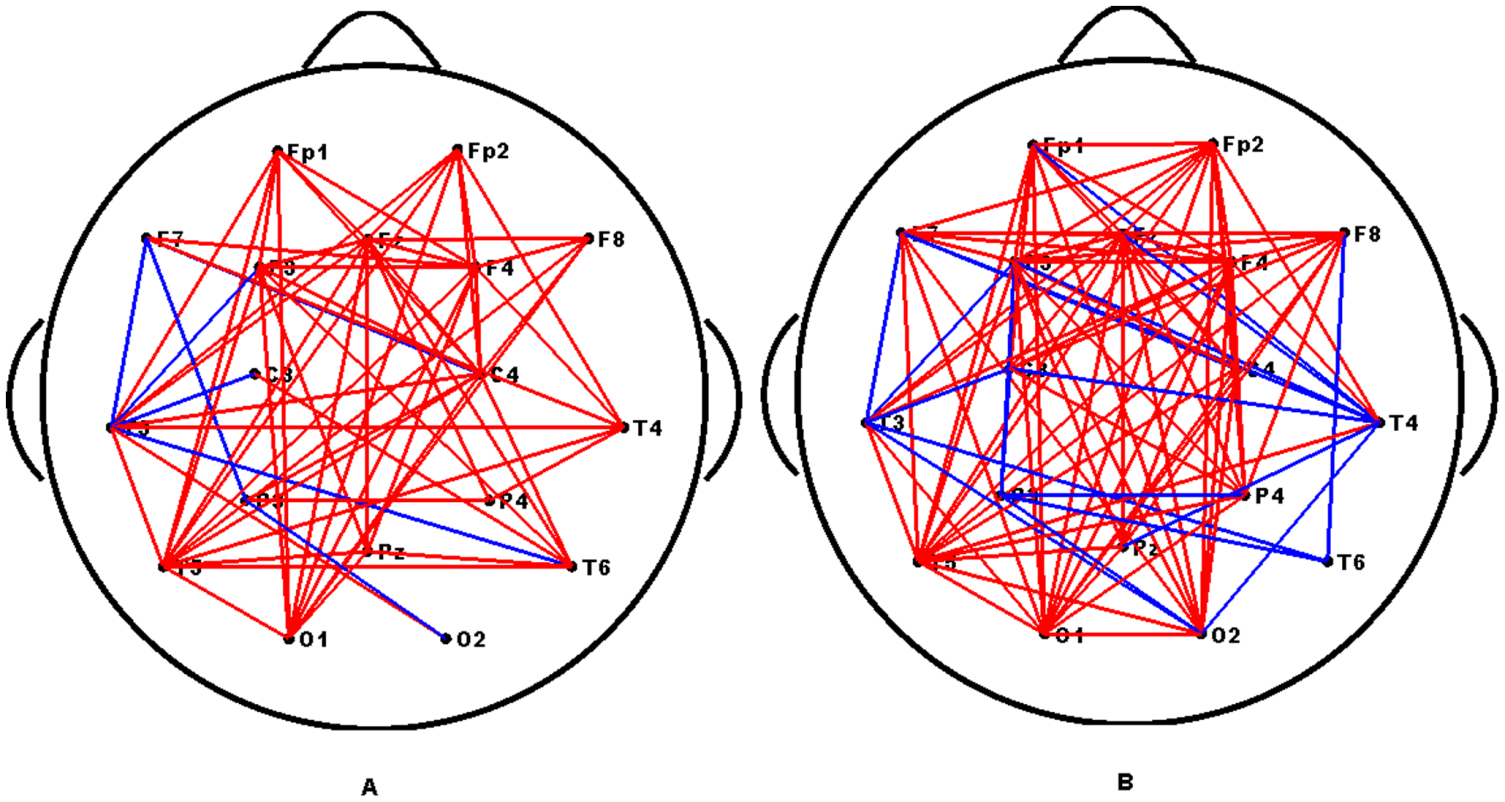
Nodal connection differences of networks of the subject from Group 1 and Group 2. (A) 12.5 Hz, (B) 16.6 Hz. The red and blue lines indicate the nodal connection weights in the networks of Group 1 show significantly increased and decreased compared with those of Group 2, respectively. The results were produced using permutation testing, *p*<0.05.

Nodal connection differences of networks of the subject from Group 1 and Group 2 are showed in Fig. 7. In comparison with Group 2, the networks of Group 1 contain more connections with increased connection weights (as shown with red lines), and they also have some connections with decreased connection weights (as shown with blue lines) under both frequencies. Most of the connections with increased weights were long-range. In addition, we found an increased number of long-range connections in the networks of Group 1 at 16.6 Hz compared with 12.5 Hz. This finding suggests that the long-range connections are crucial in efficient global information integration and processing. They play a crucial role in maintaining a short path length in a network, which promotes effective interactions between and across different regions [35], [43]. The larger SSVEP may mainly depend on these increased connectivity strengths of long-range connections. Our findings may suggest that a larger SSVEP is more likely related to a short path length. These results may coincide with the finding that the primary significant links are the long-range connections.

### Does the Skull Thickness or Noises Result in our Findings?

One possible reason for the differences between subjects in both SSVEP and graph measures could be from variations in the overall EEG signal quality or skull thickness.If this were the case, we would observe that connections that significantly correlated with the SNRs were distributed across the majority of the brain regions (Fig. 4), and the difference between the high SNR and low SNR groups of subjects must also distribute across most of the brain regions (Fig. 7). However, as shown in Fig. 4, the significant connections are primarily long-range connections from the occipital region to the frontal region. In addition to the positively correlated connections, there were also some sparse connections showing negative correlation with the SNRs (Figs. 4 and 5). Concurrently, Fig. 7 shows that the long-range connections may be the main factors driving the observed differences between the two groups of subjects. Accordingly, we cannot attribute either the differences or the correlations between the SNRs and networks measurements to variations of the skull thickness or noise levels. In fact, as the closely spaced electrode pairs were subject to significant volume conduction effects [25], [27], we limited our selection to 18 standard electrodes for the nodes used to construct the networks to reduce volume conduction.

### The SSVEPs are Multiple Sources Responses

Previous studies confirmed that SSVEPs involve both local and distant widely distributed brain regions [9]. These results may explain why we found that SSVEP correlated with the local metrics, i.e., clustering coefficient and local efficiency, and the global metrics of characteristic path length and global efficiency. However, our findings further show that more efficient functional networks composed of locally and non-locally distributed brain regions are related to larger responses when processing the same sensory inputs. Meanwhile, the present results provide new insights into the origination of SSVEP from spatially distributed regions, not just a single dipole source within the occipital region [9], [20].

### Methodological Considerations and Potential Topics for the Future

Volume conduction is a major factor when calculating the scalp EEG coherence, as EEG coherence is only meaningful for widely spaced electrode pairs [25], [27]. In this study, we attempted to reduce this influence by choosing 18 widely spaced electrodes to cover the main regions for recording SSVEP. Another important factor in calculating coherence is the reference. In this work, we adopted the novel zero-reference to avoid a reference effect [24], [44].

In this study, we focused on a weighted network analysis with the graph theories and calculated all the properties based on a fully connected network after reducing some spurious edges. We did not take the unweighted networks into consideration, as these types of networks could not reflect the physical information related to the graph [45], and because some studies also showed similar results when using the weighted and unweighted networks [46].

In this paper, we used two frequencies and eleven subjects to determine strong correlation between SSVEP and the global topological properties. In future studies, we plan to explore more frequencies and adopt a larger number of subjects to investigate the consistency of the results. Meanwhile, one of the great challenges in BCI research is BCI illiteracy [47]. It would be of significant value to determine if the network parameters can aid in screening subjects prior to BCI application using the resting-state recording data Additionally, effective connectivity would also be conducted to investigate the properties of the directed network.

In this study, subjects were not asked to perform cognitive task. Thus, potential future research may combine SSVEP with network analysis during tests of various cognitive activities, such as visual attention and working memory. Using this new analysis method to study change in SSVEP during a cognitive process may provide new avenues for both cognitive and clinical research.

## Conclusions

In this study, we explored the mechanism of SSVEP using brain networks analysis methods. The main findings were that larger SSVEP responses correlate with high mean functional connectivity and more efficient topological organizations of brain networks entrained by the stimulus. We found that the connections in which connection strengths significantly correlated with SSVEP were primarily long-range connections and that these long-range connections may contribute to the differences between subjects. We also found that the connections that did not non-significantly correlate with SSVEP still contribute to SSVEP generation, although the connections that directly correlated with SSVEP may be beneficial factors. Together, these results suggest that the network topology substantially contribute to the generation of SSVEP. Therefore, these results may provide insight into the mechanism of SSVEP and open opportunities for future studies. Functional brain network analysis based on graph theories may provide a novel approach for probing the underlying mechanism of SSVEP-BCI and invite new insights into relevant cognitive and clinical studies using SSVEP.
